# Selective CO_2_ Electroreduction to Ethylene and Multicarbon Alcohols via Electrolyte‐Driven Nanostructuring

**DOI:** 10.1002/anie.201910155

**Published:** 2019-10-08

**Authors:** Dunfeng Gao, Ilya Sinev, Fabian Scholten, Rosa M. Arán‐Ais, Nuria J. Divins, Kristina Kvashnina, Janis Timoshenko, Beatriz Roldan Cuenya

**Affiliations:** ^1^ Department of Interface Science Fritz Haber Institute of the Max Planck Society 14195 Berlin Germany; ^2^ Department of Physics Ruhr-University Bochum 44780 Bochum Germany; ^3^ Rossendorf Beamline at ESRF—The European Synchrotron CS40220 38043 Grenoble Cedex 9 France; ^4^ Helmholtz Zentrum Dresden-Rossendorf (HZDR) Institute of Resource Ecology PO Box 510119 01314 Dresden Germany

**Keywords:** adsorbed halides, CO_2_ electroreduction, copper(I), electrolyte-driven nanostructuring, multicarbon products

## Abstract

Production of multicarbon products (C_2+_) from CO_2_ electroreduction reaction (CO_2_RR) is highly desirable for storing renewable energy and reducing carbon emission. The electrochemical synthesis of CO_2_RR catalysts that are highly selective for C_2+_ products via electrolyte‐driven nanostructuring is presented. Nanostructured Cu catalysts synthesized in the presence of specific anions selectively convert CO_2_ into ethylene and multicarbon alcohols in aqueous 0.1 m KHCO_3_ solution, with the iodine‐modified catalyst displaying the highest Faradaic efficiency of 80 % and a partial geometric current density of ca. 31.2 mA cm^−2^ for C_2+_ products at −0.9 V vs. RHE. Operando X‐ray absorption spectroscopy and quasi in situ X‐ray photoelectron spectroscopy measurements revealed that the high C_2+_ selectivity of these nanostructured Cu catalysts can be attributed to the highly roughened surface morphology induced by the synthesis, presence of subsurface oxygen and Cu^+^ species, and the adsorbed halides.

## Graphical abstract

**Electrolyte‐driven nanostructuring** can be used to synthesize highly selective Cu catalysts for CO_2_ electroreduction to multicarbon products (80 % Faradaic efficiency). Operando spectroscopy measurements showed that apart from enhanced roughness from the pre‐treatment, the subsurface oxygen and Cu^+^ species and the adsorbed halides favor C−C coupling, leading to high selectivity for ethylene, ethanol, and n‐propanol.

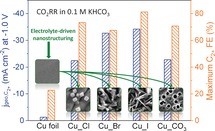

## Introduction

The electrochemical production of fuels and chemical feedstocks from CO_2_ and water using the electricity derived from renewable energy holds promise as a sustainable process that might help to mitigate some of our current energy and climate challenges. CO_2_ electroreduction reaction (CO_2_RR) to multicarbon hydrocarbons and oxygenates (C_2+_) with high energy density is highly desirable, but is severely limited by the slow kinetics of multiple proton and electron transfer steps during C−C coupling.^1^, ^2^, ^3^, ^4^ Cu, among the studied metals, is the only one producing hydrocarbons and alcohols in considerable amounts. However, polycrystalline Cu usually suffers from high overpotential and low C_2+_ selectivity.^5^ The formation of C_2+_ products during CO_2_RR has been found to be extremely sensitive to the catalyst structure.^1^, ^6^, ^7^, ^8^ Therefore, nanostructured electrocatalysts capable of efficient generation of multicarbon products from CO_2_RR might be developed through rational design.

It is well‐known that the activity and selectivity of CO_2_RR catalysts strongly depend on the precise control of their structure, such as the content of defects,^9^ subsurface oxygen or Cu^+^ species,^10^, ^11^, ^12^, ^13^, ^14^, ^15^ the specific shape of the nanocrystals,^16^, ^17^, ^18^ or the surface composition and atomic ordering in bimetallic nanostructures.^19^, ^20^ Previous experimental and theoretical studies demonstrated that Cu(100) is the most favorable crystal orientation for the C−C coupling process.^21^, ^22^, ^23^ However, the surface of Cu electrodes under electrochemical environments often undergoes reconstructions induced by applied potentials,^24^, ^25^, ^26^ the intermediates formed during CO_2_RR,^27^ as well as specifically adsorbed anions.^28^, ^29^, ^30^ On the other hand, some anions either present in the electrolyte^12^, ^31^, ^32^ or adsorbed on the electrode surface,^33^ have been shown to play a vital role in the dynamic evolution of the catalyst structure under reaction conditions as well as the activity and selectivity of CO_2_RR. These important findings might be used in the design and development of new electrodes via electrochemical modifications.

The morphology and composition of electrochemically synthesized catalysts is strongly affected by the applied potential and electrolyte employed.^34^ Herein we report an electrolyte‐driven nanostructuring strategy for the facile synthesis of highly selective CO_2_RR catalysts. The nanostructured Cu catalysts synthesized in the presence of specific anions can selectively convert CO_2_ to ethylene and multicarbon alcohols in aqueous 0.1 m KHCO_3_ solution, with the KI‐pretreated catalyst displaying the highest FE of about 80 % and partial current density of about 31.2 mA cm^−2^ for C_2+_ products at −0.9 V vs. RHE. The high C_2+_ selectivity of these nanostructured Cu catalysts is attributed to their rough morphology, the presence of subsurface oxygen, Cu^+^ species, and adsorbed halides on the surface.

## Results and Discussion

**Figure 1:**
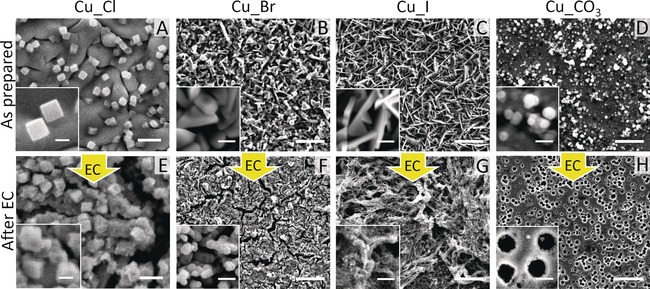
SEM images of Cu_Cl, Cu_Br, Cu_I, and Cu_CO_3_ samples before and after 1 h of CO_2_RR at −1.0 V vs. RHE in a CO_2_‐saturated 0.1 m KHCO_3_ solution. The scale bars in the main images and insets are 1 μm and 200 nm for the Cu_Cl sample (A,E), 5 μm and 500 nm for Cu_Br (B,F), Cu_I (C,G), and Cu_CO_3_ samples (D,H).

Nanostructured Cu catalysts were synthesized by cycling electropolished Cu foils in different 0.1 m potassium salt solutions (between 0.3 and 2.2 V vs. RHE) and were denoted by Cu_X (X=Cl, Br, I) and Cu_CO_3_, respectively. Additional details on the synthesis parameters are shown in the Supporting Information, Table S1. Figure 1 and the Supporting Information, Figure S1 show scanning electron microscopy (SEM) images of these samples as‐prepared, after immersion in 0.1 m KHCO_3_ solution, as well as after 1 h of CO_2_RR at −1.0 V vs. RHE. Cu nanocubes with an edge size of 250–300 nm are formed on the surface of the Cu_Cl (Figure 1 A) sample as we discussed previously.^18^ In clear contrast, the Cu_Br and Cu_I samples show a faceted crystal morphology characterized by flatter larger structures for Cu‐Br (Figure 1 B) and needle‐like shapes for Cu_I (Figure 1 C). A composition of CuBr and CuI was confirmed by energy‐dispersive X‐ray spectroscopy (EDX; Supporting Information, Table S2). The Cu_CO_3_ sample shows particles dispersed on the underlying Cu surface (Figure 1 D). After the former electrolyte‐driven surface nanostructuring, when the different samples are subsequently immersed in the same 0.1 m KHCO_3_ solution for 30 min before applying any potential, the edges of CuBr and CuI crystals as well as nanocubes in the Cu_Cl sample become slightly roughed (Supporting Information, Figure S1), and an increased oxygen content and decreased halide content are observed due to the slow decomposition of the Cu halide (CuX) in the aqueous solution (Supporting Information, Table S2). The highest halide content of the three Cu_X samples is consistent with the highest stability of CuI among the possible CuX compounds that could be formed.^33^ The crystalline structure of the copper halide layer in the as prepared state was further confirmed by grazing incidence X‐ray diffraction (GI‐XRD) measurements, as shown in the Supporting Information, Figure S2. All three halide‐modified Cu samples consist of their copper halide phases, with the exception of the Cu_Cl sample, which also includes minor Cu_2_O peaks owing to the instability of CuCl.

More interestingly, these samples show very different morphologies after 1 h of CO_2_RR, although most of the halides and carbon atoms have been removed during the reaction (Supporting Information, Table S2). Numerous particles with average size of 220±60 nm are formed on the surface of the Cu_Br sample (Figure 1 F), while the Cu_I sample shows a rough surface (Figure 1 G), with a very high roughness factor determined by measuring the double‐layer capacitance (Supporting Information, Table S3). The particles on the surface of Cu_CO_3_ are removed during CO_2_RR, leading to a porous Cu surface with average pore size of 430±130 nm (Figure 1 H).

**Figure 2:**
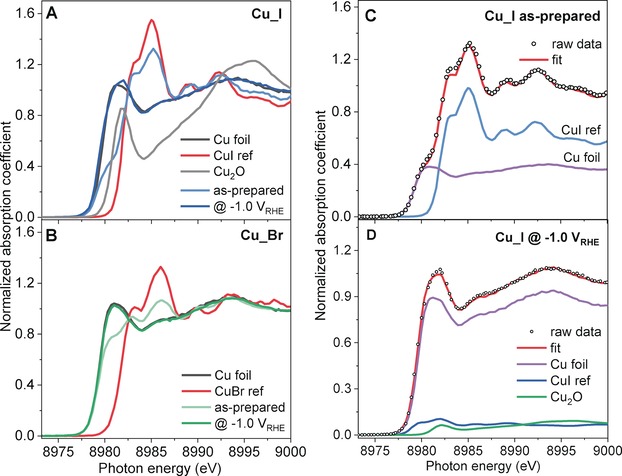
HERFD‐XANES spectra of the Cu_I (A) and Cu_Br (B) samples in the as‐prepared state and measured under operando CO_2_RR conditions in 0.1 m KHCO_3_ after 1 h of CO_2_RR at −1.0 V vs. RHE. Reference spectra of bulk Cu, CuI, CuBr, and Cu_2_O are also plotted. Linear combination analysis (LCA) of operando HERFD‐XANES spectra of the Cu_I sample measured in the as‐prepared state (C) and during CO_2_RR at −1.0 V vs. RHE (D) are shown. The corresponding subspectral components needed to fit the data (metallic Cu, CuI, and Cu_2_O) are scaled according to their weighting parameters.

To monitor the evolution of the chemical state and local environment of Cu during CO_2_RR we conducted operando X‐ray absorption spectroscopy (XAS) measurements. While extended X‐ray absorption fine‐structure spectroscopy (EXAFS) data measured in total fluorescence yield mode did not show any significant difference from the metallic structure apart from a highly defective structure (Supporting Information, Figures S3–S7, Table S4), high‐energy resolution fluorescence detected X‐ray near edge structure (HERFD‐XANES) spectra are more sensitive to the chemical state and coordination environment of Cu under reaction conditions.^35^, ^36^ Figure 2 shows the Cu K‐edge HERFD‐XANES spectra of the as prepared Cu_X samples as well as data from the same samples obtained during CO_2_RR at −1.0 V vs. RHE along with the reference spectra of bulk Cu, Cu_2_O, CuI, and CuBr. The spectra suffer from significant self‐absorption effects, but a normalization was used here to have equal self‐absorption in the reference spectra and halide‐ and carbonate‐modified samples. The spectra of the as‐prepared Cu_I and Cu_Br samples show distinctive features of CuI (Figure 2 A; Supporting Information, Figure S8) and CuBr (Figure 2 B). A broad pre‐edge feature between 8979 and 8981 eV, most likely corresponding to metallic Cu, is also detected for the Cu_I sample. The more intense pre‐edge feature at 8981 eV in the Cu‐Br sample points out a higher amount of metallic Cu. The as‐prepared Cu_Cl and Cu_CO_3_ samples on the other hand show the presence of a dominant metallic Cu component (pre‐edge feature at 8981 eV; Supporting Information, Figures S9, S10, respectively). A slightly more intense feature at 8986 eV and lower intensity of the above mentioned metallic feature in the as‐prepared Cu_Cl indicate the presence of CuCl species. Finally, there is no clear evidence of cationic Cu in the as‐prepared Cu_CO_3_ sample.

HERFD‐XANES spectra of all four samples measured under CO_2_RR conditions after 1 h of activation indicate nearly complete reduction of Cu, showing a close resemblance to the reference spectrum of the Cu foil. An exception however is the Cu_I sample measured at −1.0 V vs. RHE, which has the position of the pre‐edge feature shifted to the energy typical for Cu_2_O. To support the described qualitative observations, linear combination analysis (LCA) of the XANES spectra was carried out using various combinations of reference spectra as basis sets (Figure 2 C,D; Supporting Information, Figures S9–S11). According to the LCA analysis, the as‐prepared Cu_X samples contain Cu^+^ species (CuI, CuBr, CuCl, and/or Cu_2_O), the relative content of which follows the sequence Cu_I> Cu_Br > Cu_Cl (63, 32, and 24 at % correspondingly; Supporting Information, Table S5). Notably, no CuO_*x*_ could be detected in the as‐prepared Cu_I and Cu_Br samples, while the Cu_Cl and Cu_CO_3_ samples contained correspondingly Cu_2_O (14 at %) and CuO (3 at %). As mentioned above, the Cu_CO_3_ sample is fully reduced after 1 h under CO_2_RR, while Cu_I, Cu_Br, and Cu_Cl show the presence of Cu_2_O in the amounts of 8, 3 and 1 at %, respectively, albeit the latter two numbers are within the experimental error. The reduced Cu_I sample still contained ca. 7 at % of residual CuI. These results also reveal the higher sensitivity of HERFD‐XANES measurements acquired in grazing configuration to Cu_2_O and CuI species as compared to GI‐XRD which only shows metallic Cu peaks after CO_2_RR (Supporting Information, Figure S2).^36^, ^37^

**Figure 3:**
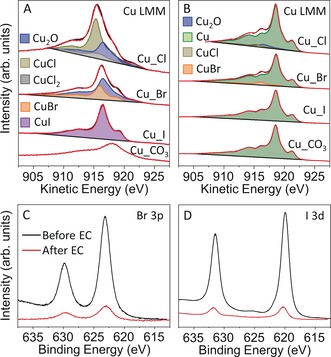
Quasi in situ Cu Auger LMM XPS spectra of Cu_CO_3_, Cu_Br, and Cu_I before (A) and after (B) 1 h of CO_2_RR at −1.0 V vs. RHE in a CO_2_‐saturated 0.1 m KHCO_3_ solution. Br 3p and I 3d XPS spectra of the Cu_Br (C) and Cu_I (D) measured before and after CO_2_RR are also shown.

Quasi in situ X‐ray photoelectron spectroscopy (XPS) measurements were performed to probe the chemical state and composition of the surface of the Cu catalysts during CO_2_RR. The electrochemical cell was directly attached to the XPS analysis system and the sample transfer was conducted in UHV. The Cu_X and Cu_CO_3_ samples were measured with XPS in the as‐prepared state and after 1 h of CO_2_RR at −1.0 V vs. RHE as shown in Figure 3 and the Supporting Information, Figures S12 and S13. Among the Cu_X samples, the Cu_I sample shows an almost pure CuI surface in its as prepared state, while the surface of the Cu_Br is composed of Cu_2_O (65 at %) and CuBr (35 at %), and that of Cu_Cl is composed of Cu_2_O (31 at %), CuCl (56 at %) and CuCl_2_ (12 at %). The composition difference is also consistent with the relative stability of the three Cu halides.^33^ However, the Cu_CO_3_ sample has a starting Cu oxidation state of Cu^2+^ in the form of CuO and CuCO_3_. After CO_2_RR, most of the Cu^+^ and Cu^2+^ species in the as prepared samples were reduced to metallic Cu. However, a small amount of CuBr still survived (Figure 3 B). Moreover, considerable amounts of halides (Figure 3 C,D) were observed on the Cu surface before but also after the reaction and subsequent in situ rinsing in water. The metallic Cu surface of the Cu_I sample seen by XPS, together with the Cu_2_O and CuI species revealed by the more bulk‐sensitive XANES measurements (Figure 2), indicate the presence of subsurface oxygen and Cu^+^ species in the halide‐derived CO_2_RR Cu catalysts.

**Figure 4:**
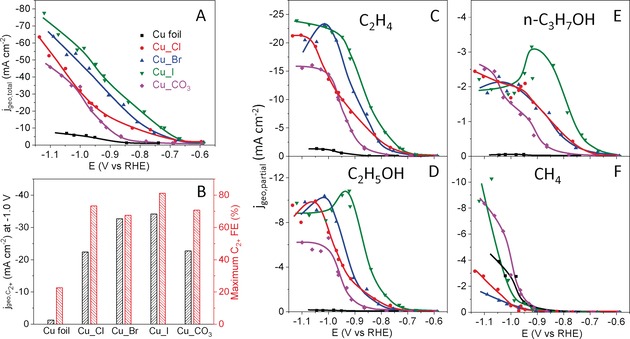
Total geometric current density (A), total Faradaic efficiency and geometric partial current density of (B) C_2+_ products, and partial geometric current densities of C) C_2_H_4_, D) C_2_H_5_OH, E) n‐C_3_H_7_OH, and F) CH_4_ as a function of the applied potential after 1 h of CO_2_RR in a CO_2_‐saturated 0.1 m KHCO_3_ solution.

The catalytic activity and selectivity of the nanostructured Cu catalysts were obtained by performing chronoamperometry measurements in a CO_2_‐saturated 0.1 m KHCO_3_ solution. All the nanostructured Cu catalysts show significantly higher geometric current density than an electropolished Cu foil (EP_Cu), and Cu_I shows the highest current density in the measured potential range (Figure 4 A). However, when the current densities were normalized by the electrochemically active surface area (ECSA), the three halide‐modified Cu catalysts show similar specific activity (Supporting Information, Figure S17A). Therefore, the high surface area (thus high density of surface reactive sites) of the roughened Cu catalysts plays a very important role in the significantly improved apparent activity compared to a flat Cu surface. Nevertheless, apart from the roughness, the increased activity over the nanostructured Cu catalysts versus EP_Cu could also be ascribed to defects, their porous structures, and the presence of adsorbed halide species, Cu^+^ or subsurface oxygen species, as we observed by XPS (Figure 3) and XANES (Figure 2).

The total FEs of C_2+_ products are shown in Figure 4 B and the Supporting Information, Figure S14, and FEs for each individual product are shown in the Supporting Information, Figure S15. The highest C_2+_ FE of about 80 % was achieved at −0.9 V vs. RHE over Cu_I, while the C_2+_ FEs of about 66–73 % at −1.0 V vs. RHE were obtained over Cu_Cl, Cu_Br, and Cu_CO_3_. The C_2+_ FEs of all nanostructured Cu catalysts were 3‐fold higher than those of the EP_Cu sample (ca. 20 %) at −0.9 V vs. RHE. At more negative potentials (<‐1.0 V vs. RHE), all the catalysts show decreased C_2+_ FE and increased H_2_ FE, which is due to CO_2_ mass‐transport limitations. The potential‐dependent partial current densities of the various CO_2_RR products are shown in Figure 4 C–F and the Supporting Information, Figures S16, S17. Cu_I showed the highest partial current densities for ethylene, ethanol, and n‐propanol. Compared to the previously reported systems, where EP_Cu was studied in an electrolyte mixture containing KX (X=Cl, Br, I) and KHCO_3_,^17^ our new nanostructured catalytic systems (synthesized by cycling the Cu foil in KX followed by washing in water) avoided the complexity of the co‐existence of structural and chemical electrolyte effects since all CO_2_RR measurements were done in KHCO_3_. Furthermore, higher activity, lower onset potential and higher C_2+_ selectivity were obtained for the present electrolyte pre‐nanostructured catalysts as compared to the case where iodine was added to the electrolyte during the reaction (Supporting Information, Figure S18). Although the Cu_CO_3_ sample showed high partial current density for C_2+_, its methane partial current density was also higher, comparable to that of EP_Cu. The simultaneously favorable production of ethylene and methane over the porous Cu_CO_3_ is slightly different from that reported for Cu foam catalysts, which simultaneously favored the production of ethylene and ethane, which is probably due to the smaller pore size (430 nm) in our Cu_CO_3_. Overall, the iodine modified Cu catalyst (Cu_I) synthesized via electrolyte‐driven nanostructuring showed a high C_2+_ geometric current density of about 31.2 mA cm^−2^ at −0.9 V vs. RHE (Supporting Information, Figure S19), superior to previously reported Cu catalysts (Supporting Information, Table S6).

**Figure 5:**
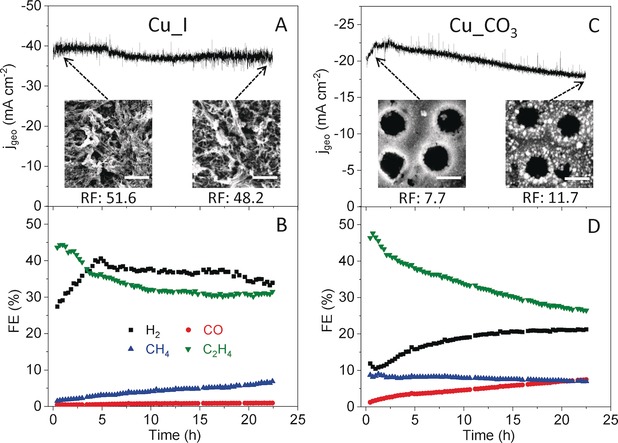
Time‐dependent geometric current densities and Faradaic efficiencies of gas products for the Cu_I sample at −0.9 V vs. RHE (A, B) and the Cu_CO_3_ sample at −0.95 V vs. RHE (C, D) in a CO_2_‐saturated 0.1 m KHCO_3_ solution. The insets in (A) and (C) are SEM images of the Cu_I and Cu_CO_3_ samples after 1 h and after 22 h of CO_2_RR. Scale bars: 5 μm (A) and 500 nm (C). Roughness factors (RF) after 1 h and after 22 h of CO_2_RR are also indicated.

Stability tests were carried out for the Cu_I and Cu_CO_3_ samples at potentials for which the highest C_2+_ FEs were detected (Figure 5). The current density of Cu_I remained almost stable within our 22 h test, while a 20 % decrease was observed for the Cu_CO_3_ sample. Furthermore, a decrease in the C_2_H_4_ FE was observed for both samples, although in the case of the Cu_I this took place only during the first 5 h, becoming stable subsequently. The decrease in the C_2_H_4_ FE was accompanied by an increase in methane FE for the Cu_I sample (a fast decrease in C_2_H_4_/CH_4_ FE ratio; Supporting Information, Figure S20) and of CO and H_2_ FE for Cu_CO_3_. However, the morphology (inserts in Figure 5 A) and roughness (Supporting Information, Table S3) of Cu_I after the stability test were similar to those after 1 h of CO_2_RR, suggesting that the increased production of CH_4_ (or CO) at the expense of C_2_H_4_ was not caused by morphological changes, but most likely by the gradual depletion of subsurface oxygen species^38^, ^39^, ^40^ and Cu^+^ species as well as the loss of adsorbed iodine ions in the Cu_I sample. In clear contrast, a large number of NPs were formed during CO_2_RR on the surface of the Cu_CO_3_ sample (inserts in Figure 5 C) and the roughness increased accordingly (Supporting Information, Table S3). The presence of these new low‐coordinated sites in the form of NPs is expected to favor the formation of CO and H_2_ 41 and could also lead to the deactivation of the Cu_CO_3_ sample.

Although the activity, selectivity, and stability of CO_2_RR catalysts are determined by multiple complex factors such as roughness, defects, shape, and oxidation state, the present data feature that the C−C coupling process over the Cu_X samples, especially Cu_I, is strongly related to the presence and stabilization of Cu^+^ species as well as of the adsorbed halides, as confirmed by operando HERFD‐XANES (bulk‐sensitive) and quasi in situ XPS measurements (surface sensitive). We found a positive correlation between the production of C_2+_ and the amount of Cu^+^ species in the halide‐modified Cu catalysts in the following order: Cu_I> Cu_Br > Cu_Cl. Previous theoretical studies predicted that subsurface oxygen as well as the presence of a Cu^+^/Cu^0^ interface plays a crucial role in CO_2_ activation and CO dimerization, ultimately resulting in higher C_2+_ selectivity.^11^, ^42^ Interestingly, the adsorbed halides are known to bind more strongly to the oxidized Cu surface^12^, ^31^ and to facilitate the formation and stabilization of the intermediates during CO_2_RR required to obtain C_2+_ products. On the other hand, the Cu_CO_3_ sample with only metallic Cu species under reaction conditions, showed higher CH_4_ selectivity than all Cu_X samples. The latter is probably attributed to its nanoporous structure.^43^ At the end, we should also highlight the role of the high ECSA of the present nanostructured Cu catalysts.^44^ Apart from having a higher surface area, a drastic increase in the ECSA during nanostructuring a flat Cu surface is usually coupled with the creation of highly reactive surface sites such as defects and low‐coordinated sites. These surface sites might be more favorable for C−C coupling during CO_2_RR,^1^, ^45^ not only improving the apparent activity but also helping to tune the selectivity towards multicarbon products. In this work we were able to modify the surface morphology and its composition and chemical state (Cu^+^) via an electrolyte‐driven nanostructuring pre‐treatment strategy, which was found to lead to enhanced C_2+_ selectivity.

## Conclusion

We have presented an electrolyte‐driven nanostructuring strategy for the facile synthesis of CO_2_RR electrocatalysts highly selective to C_2+_ products. The proposed synthesis not only leads to strong morphological modifications of the sample surface, but also to the presence of residual halides and cationic Cu species. These Cu electrocatalysts can selectively convert CO_2_ into ethylene and multicarbon alcohols in a KHCO_3_ solution, with the iodine‐modified catalysts showing the highest FE for C_2+_ of about 80 % and partial geometrical current density of about 31.2 mA cm^−2^ at −0.9 V vs. RHE. The superior C_2+_ selectivity of the halide‐modified Cu catalysts was attributed to their rough surface morphology combined with electronic and chemical effects arising from the stabilization of subsurface oxygen as well as Cu^+^ species and adsorbed halides on the surface. Cu_CO_3_ shows both high C_2+_ and methane selectivity, which is attributed to its particular nanoporous structure. Stability tests suggested that the gradual depletion of subsurface oxygen/Cu^+^ species and the increased number of low‐coordinated sites formed under reaction conditions are behind the distinct catalytic performance of the halide‐ and carbonate‐modified Cu catalysts, respectively. This work provides new insights required for the design of highly active C_2+_‐selective CO_2_RR catalysts.

## Experimental Section

Cu_Cl, Cu_Br, Cu_I, and Cu_CO_3_ catalysts were prepared by electrochemically cycling an electropolished Cu foil in 0.1 m KCl, KBr, KI, and K_2_CO_3_ solutions with triangular potential scans at a rate of 500 mV s^−1^, respectively. During each cycle, the potential was held at the negative (E_1_) and positive (E_2_) limits for 5 and 10 s, respectively. The cycled Cu catalysts were prepared with the indicated potential ranges and number of cycles as shown in the Supporting Information, Table S1. For other experimental details, including operando and ex situ characterizations and electrochemical measurements, see the Supporting Information.

## Conflict of interest

The authors declare no conflict of interest.

## Supporting information

As a service to our authors and readers, this journal provides supporting information supplied by the authors. Such materials are peer reviewed and may be re‐organized for online delivery, but are not copy‐edited or typeset. Technical support issues arising from supporting information (other than missing files) should be addressed to the authors.

Supplementary
