# Supplementary material for: Selective CO2 Electroreduction to Ethylene and Multicarbon Alcohols via Electrolyte‐Driven Nanostructuring
Source: Angew Chem Int Ed Engl. 2019 Oct 8;58(47):17047–53. doi: 10.1002/anie.201910155 (PMC6899694; doi:10.1002/anie.201910155)
Supplement: Supplementary file 1 — Supplementary [file ANIE-58-17047-s001.pdf]

Angewandte Chemie
Eine Zeitschrift der Gesellschaft Deutscher Chemiker

Supporting Information

Selective CO_2 Electroreduction to Ethylene and Multicarbon Alcohols via Electrolyte-Driven Nanostructuring

*Dunfeng Gao, Ilya Sinev, Fabian Scholten, Rosa M. Arán-Ais, Nuria J. Divins, Kristina Kvashnina, Janis Timoshenko, and Beatriz Roldan Cuenya**

anie_201910155_sm_miscellaneous_information.pdf

Catalysts synthesis

Commercial Cu foils (Advent Research Materials Ltd., 99.995%, 0.125 mm thick) were first cleaned with acetone and ultrapure water (18.2 MΩ) in an ultrasonic bath, and then electropolished in phosphoric acid (VWR, 85 wt%) at 3 V *versus* a titanium foil for 5 min. Cu_Cl, Cu_Br, Cu_I and Cu_CO_3 catalysts were prepared by electrochemically anodizing an electropolished Cu foil in 0.1 M KCl (Sigma-Aldrich, 99%), KBr (Sigma-Aldrich, 99%), KI (Sigma-Aldrich, 99%) and K_2CO_3 (VWR, 99.7%) solutions with triangular potential scans at a rate of 500 mV s^{-1}, respectively. During each cycle, the potential was held at the negative (E_1) and positive (E_2) limits for 5 and 10 s, respectively. The cycled Cu catalysts were prepared with the indicated potential ranges and number of cycles as shown in Table S1. After the former treatments, the samples were rinsed in ultrapure water to remove the electrolytes.

SEM characterization

The morphology of the nanostructured Cu catalysts was investigated by SEM using a Quanta 200 FEG microscope from FEI with a field emitter as the electron source. The images were acquired using a secondary electron (Everhart-Thornley) detector with an acceleration voltage of 10 keV and a working distance of 10 mm. This configuration was found to yield the best balance among spatial resolution, surface sensitivity, signal to noise ratio, and field depth. A separate, liquid-N_2-cooled energy-dispersive X-ray spectroscopy (EDX) detector was employed for the elemental analysis of the samples. The error bars for the content of all elements were made on the basis of the EDX spectra from at least six different positions of two identical samples. The samples were transferred immediately to the SEM chamber after the reaction and a subsequent rinse in water in order to minimize air exposure. The amount of oxygen resulting from air exposure was negligible given that EDX, with a probing depth of ~300 nm at 10 keV, is not surface-sensitive enough.

Grazing incidence XRD characterization

The modified Cu foils in the as prepared state and after CO_2RR were investigated by grazing incidence XRD using a Bruker D8 Advance diffractometer equipped with a Lynx Eye Detector and KFL Cu 2K X ray tube. Measurements were performed in a 2θ range of 20-80 ° with a step size of 0.06 °, a collection time of 40 s per step and an incident angle of 1 °.

***Operando* TFY-XAS and HERFD-XANES**

The X-ray absorption spectra of halide- and carbonate-modified Cu foils were acquired as-prepared and under CO_2RR reaction conditions using a home-built *operando* electrochemical cell with a Pt-mesh counter electrode and an Ag/AgCl reference electrode. The samples were mounted behind a Kapton window with 1 mm thick layer of electrolyte between the sample and the window. The electrolyte (0.1 M $KHCO_3$) was circulated between the cell and a reservoir continuously purged with CO_2. Two series of XAS measurements were carried out: total fluorescence yield (TFY), covering both XANES and EXAFS

regions, and high-energy resolution fluorescence detected (HERFD) XANES. To ensure maximum sensitivity to the sample surface with the XAS technique, the angle of incidence was decreased to 10°, the lowest value allowed by the current cell geometry. As a result, the contribution of deeper sample layers unmodified during nanostructuring could be minimized, as it is illustrated in the HERFD-XANES spectra of the as-prepared Cu_I sample in Figure S5.

The Cu K-edge (8979 eV) TFY-XAS (XANES/EXAFS) spectra were recorded at beamline P65 of PETRA III synchrotron light facility (DESY) in Hamburg (Germany) using a passivated implanted planar silicon (PIPS) detector. A Si(111) double crystal monochromator detuned to 65% of intensity was used for the energy scan. The HERFD-XANES measurements were performed at the Rossendorf beamline BM20 (ROBL) of the European Synchrotron Radiation facility (ESRF), Grenoble (France). The photon energy was scanned by a double-crystal Si(111) monochromator and higher harmonics were rejected by a Pt-coated collimating mirror. Cu K-edge HERFD-XANES spectra were recorded at Cu $K_{\alpha 1}$ emission line using a spherically bent Ge crystal analyzer. The IFEFFIT software package[1] was used to process and analyze the XAS data. Athena was used for data reduction, background subtraction, self-absorption correction (for TFY spectra), and linear combination analysis (LCA). For the latter, a set of reference spectra was used that was measured in the same configuration of the beamline and crystal analyzer. For each experimental spectrum, a weighting parameter (X), representing a fractional content of the corresponding species and the energy shift (ΔE) of the reference spectra were fitted, the results are summarized in Table S5 and examples of LCA are shown in Figure 2D and Figures S5-S7. Various combinations of basis sets were tried, and those resulting in the lowest R-factor were chosen. As an additional figure of merit, the combinations requiring too high-energy shift of one or more components (by more than ±5 eV) were not accepted. Extended X-ray absorption fine-structure spectra (EXAFS) were fitted in Artemis using theoretical backscattering amplitudes and phases calculated by the FEFF6 code[2] for face-centered cubic Cu metal and cubic CuI structures[3]. Coordination numbers (CN), interatomic distances (r), Debye-Waller factors (σ^2), and energy shift (ΔE_0) were the fitting parameters.

Quasi *in situ* XPS characterization

The quasi *in situ* XPS measurements were carried out in an ultrahigh-vacuum (UHV) setup equipped with a non-monochromatic Al X-ray source (hν = 1486.6 eV) and a hemispherical electron analyzer (Phoibos100, SPECS GmbH). The Cu $2p_{3/2}$ peak corresponding to Cu_2O (932.67 eV) was used for energy alignment. The XPS analysis chamber was connected to an *in situ* electrochemical (EC) cell (SPECS GmbH). An *in situ* electrochemical (EC) cell (SPECS GmbH) was connected to the XPS analysis chamber. The potential was controlled with an Autolab potentiostat (PGSTAT 302N). The sample transfer from the EC cell to the XPS UHV chamber was performed under vacuum. For the deconvolution of the Cu LMM Auger spectra, data acquired in our laboratory from a metallic Cu^0 foil (reduced *in situ* by H_2 plasma),

commercial CuCl (Alfa Aesar, 99.999%), CuBr (Sigma-Aldrich, 99.995%), CuI (Sigma-Aldrich, 99.995%) powders, and CuO and Cu_2O foils from the literature were used as references. The Cu Auger spectra are more sensitive to the presence of Cu^+ species than the Cu 2p XPS region. In particular, they can help us to distinguish Cu_2O from metallic Cu. The analysis of the O 1s spectra is much more challenging because they are dominated by the contribution of adsorbed species not associated with Cu^+.

Electrochemical measurements

Electrochemical measurements were carried out in a gas-tight H-cell separated by an anion exchange membrane (Selemion AMV, AGC Inc.) Both, working and counter compartments were filled with 40 ml 0.1 M $KHCO_3$ (Sigma-Aldrich, 99.7%) and purged continuously with CO_2 (99.995%, 20 ml min^{-1}). 0.1 M $KHCO_3$ solution was prepared by ultrapure water and further pre-purified by Chelex 100 Resin (Bio-Rad). Prior to the measurement, the electrolyte is bubbled with CO_2 for 30 min to remove oxygen in the solution and saturate the solution (pH 6.8). A platinum gauze (MaTecK, 3600 mesh cm^{-2}) was used as counter electrode and a leak-free Ag/AgCl electrode (Innovative Instruments) as the reference electrode. The prepared samples were used as working electrode and contacted with a clamp wrapped by Kapton tape to avoid the unwanted reaction. Each fresh sample was measured with a chronoamperometric step for 1 h at each potential. The potentials were controlled with an Autolab potentiostat (PGSTAT 302N). All potentials *versus* Ag/AgCl were converted to the reversible hydrogen electrode (RHE) scale and corrected for iR drop as determined by current interrupt. The roughness factors were determined by measuring the double-layer capacitance with cyclic voltammetry between 0 and 0.25 V *vs* RHE in a CO_2-saturated 0.1 M $KHCO_3$ solution, after 1 h of CO_2 electroreduction reaction at −1.0 V *vs* RHE.

Product analysis

The gas products were analyzed by online gas chromatography (GC, Agilent 7890A) every 17 min. CO, H_2 and hydrocarbons were separated by different columns (Molecular sieve 13X, HayeSep Q and Carboxen-1010 PLOT) and quantified by a thermal conductivity detector (TCD) and flame ionization detector (FID). Carboxylates (formate and acetate) formed during electrolysis were analyzed by high-performance liquid chromatography (HPLC, Shimadzu Prominence), equipped with a NUCLEOGEL SUGAR 810 column and refractive index detector (RID). Alcohols were analyzed with a liquid GC (Shimadzu 2010 plus), equipped with a fused silica capillary column and FID. An aliquot of the electrolyte after reaction was directly injected into the HPLC and liquid GC without further treatment. The reported Faradaic efficiency (FE) and production rate were calculated based on the product distribution and current after 1 h of CO_2 electroreduction reaction at constant potentials.

Table S1. Preparation parameters of the cycled Cu catalysts

Sample	Electrolyte	Potential range between E_1 and E_2 (V *vs* RHE)		Number of cycles	Electrolyte used for CO_2 electroreduction
		E_1	E_2		
Cu_Cl	0.1 M KCl	0.4	2.0	5	0.1 M $KHCO_3$
Cu_Br	0.1 M KBr	0.4	2.0	5	
Cu_I	0.1 M KI	0.4	0.8	3	
Cu_CO_3	0.1 M K_2CO_3	0.3	2.2	10	

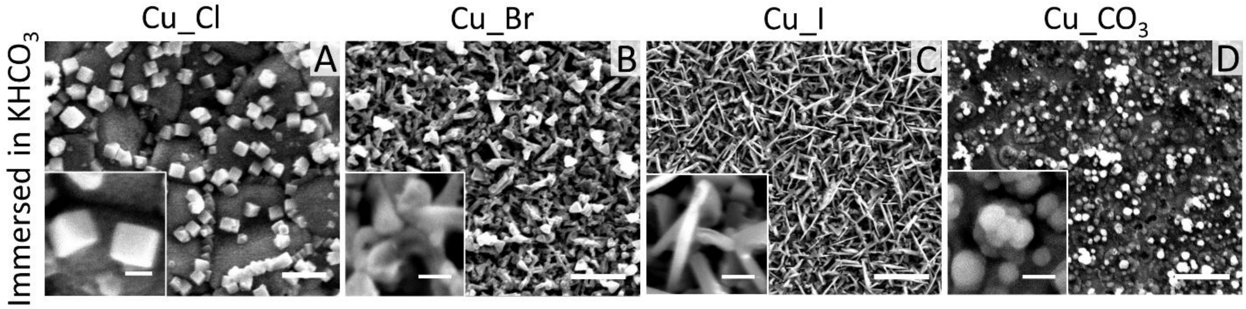

Figure S1. SEM images of the Cu_Cl, Cu_Br, Cu_I, and Cu_CO_3 samples after immersion in the electrolyte (0.1 M $KHCO_3$) for 30 min but before the reaction and in the absence of an applied potential. The scale bars in the main images and inserts are 1 μm and 200 nm for the Cu_Cl sample (A), 5 μm and 500 nm for Cu_Br (B), Cu_I (C), and Cu_CO_3 (D) samples.

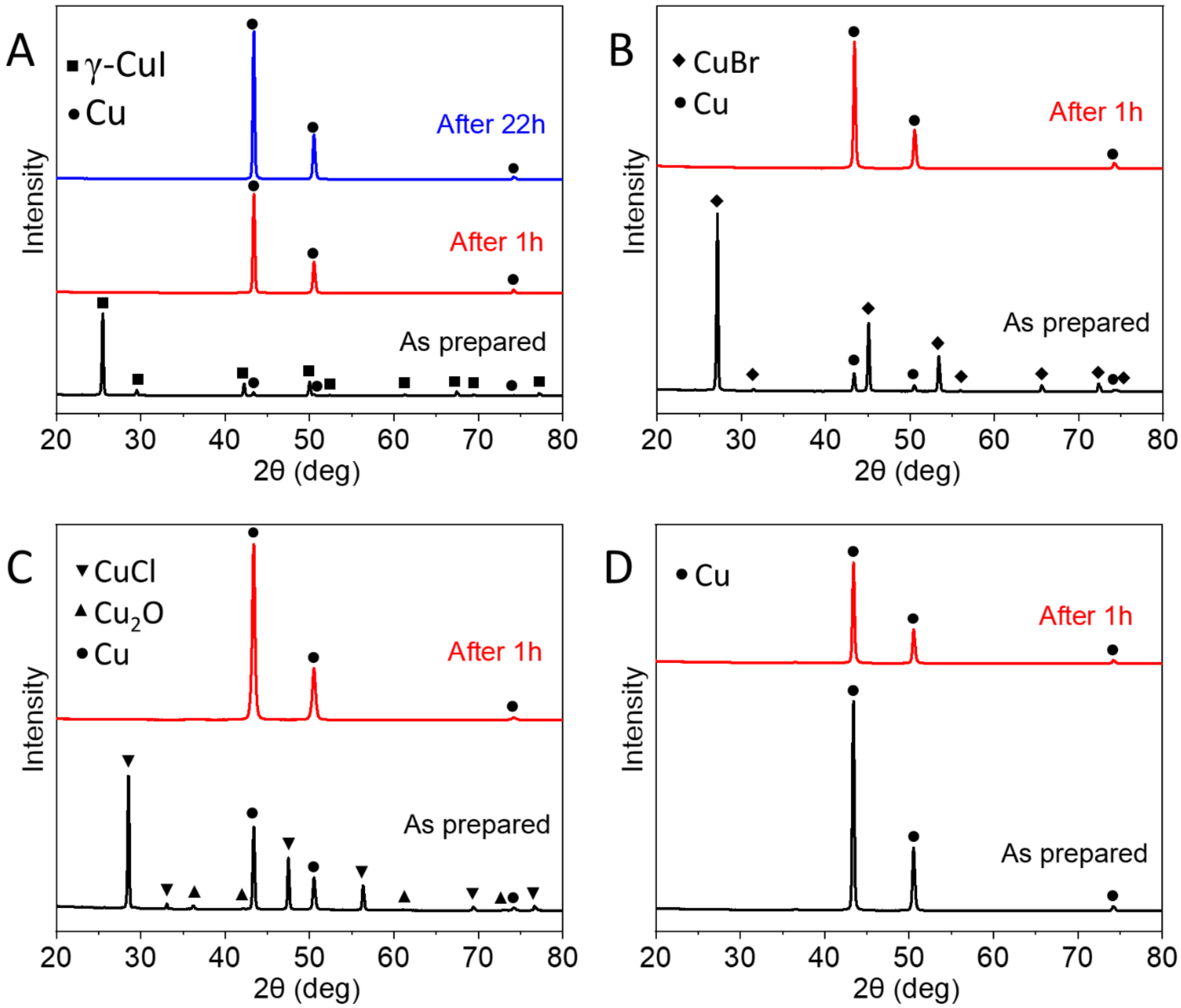

Figure S2. Grazing incidence X-ray diffraction (GI-XRD) patterns of (A) Cu_I, (B) Cu_Br, (C) Cu_Cl and (D) Cu_CO_3 samples. The angle of incidence is 1°. Cu (JCPDS 04-0836), CuI (γ-CuI, JCPDS 06-0246), CuBr (JCPDS 06-0292), CuCl (JCPDS 06-0344) and Cu_2O (JCPDS 05-0667) are marked.

Table S2. Elemental composition (atomic percentage) as determined by EDX of Cu_Br, Cu_I, Cu_CO_3, and Cu_Cl samples shown in Figure 1 and Figure S1 in the as-prepared state, after sample immersion in the different electrolytes for 30 min before applying any potential and after 1 h of CO_2 electroreduction (EC) at −1.0 V vs RHE in a CO_2-saturated 0.1 M $KHCO_3$ solution.

**Samples**		**Concentration (at%)**			
		Cu	O	Cl, Br, I, C	
Cu_Cl	As prepared_cube	55	19	Cl	26
	As prepared_foil	54	0		46
	Before EC, immersed in the electrolyte_cube	60	27		13
	Before EC, immersed in the electrolyte_foil	58	19		23
	After EC_cube	85	14		1
	After EC_foil	87	13		0
Cu_Br	As prepared	53 ± 1	2.1 ± 0.4	Br	45.2 ± 0.5
	Before EC, immersed in the electrolyte	59 ± 1	26 ± 2		15 ± 3
	After EC	95 ± 0.8	5 ± 0.8		0
Cu_I	As prepared	44 ± 2	4.3 ± 0.5	I	52 ± 1
	Before EC, immersed in the electrolyte	51 ± 1	13 ± 1		36.1 ± 0.5
	After EC	88 ± 1	11 ± 1		1.2 ± 0.3
Cu_CO_3	As prepared	45 ± 2	42 ± 2	C	13 ± 1
	Before EC, immersed in the electrolyte	45 ± 1	42 ± 2		13 ± 1
	After EC	97 ± 1	3 ± 1		0

Table S3. Roughness factors of Cu_Cl, Cu_Br, Cu_I, and Cu_CO_3 after 1 h of CO_2RR at −1.0 V vs RHE in 0.1 M $KHCO_3$, estimated through double-layer capacitance measurements. The roughness factor of an electropolished Cu foil is used as reference and defined as 1. The roughness factors of Cu_I and Cu_CO_3 are also measured after 22 h of CO_2RR stability test at −1.0 V vs RHE.

**Samples**	**Capacitance (mF cm^{-2})**	**Roughness factor**
Electropolished Cu foil	0.0258	1
Cu_Cl after 1 h	0.924	35.8
Cu_Br after 1 h	1.546	59.9
Cu_I after 1 h	1.331	51.6
Cu_CO_3 after 1 h	0.198	7.7
Cu_I after 22 h	*1.243*	*48.2*
Cu_CO_3 after 22 h	*0.303*	*11.7*

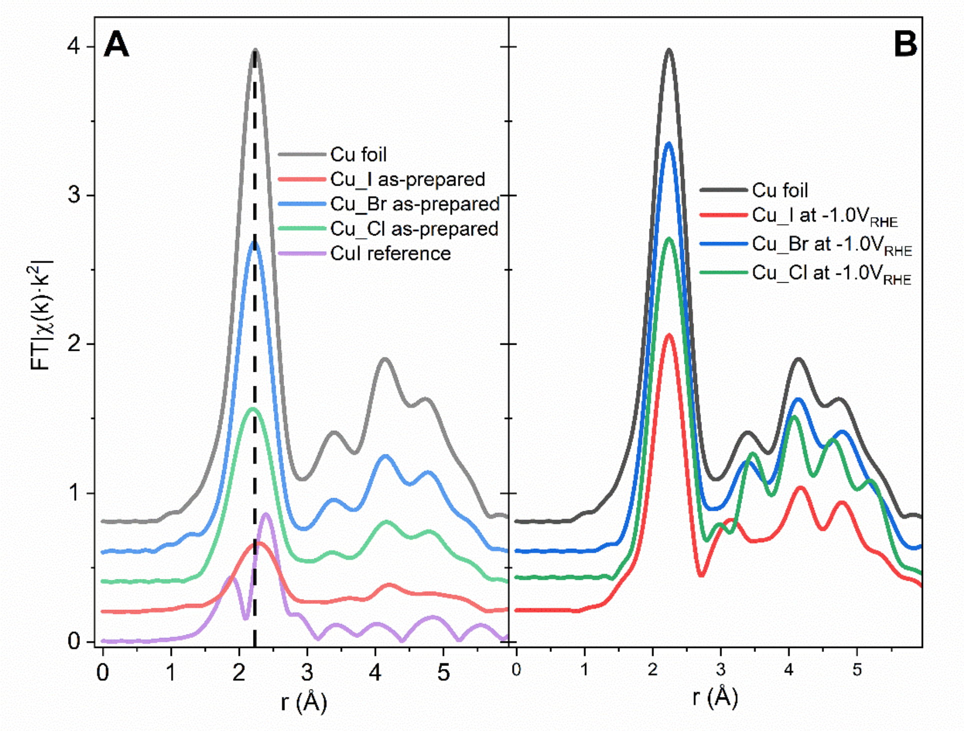

Figure S3. Cu K-edge EXAFS spectra of halide-modified Cu foils as-prepared (A) and under CO_2RR conditions after 1 h reaction at −1.0 V vs RHE in 0.1M $KHCO_3$ (B). Reference spectra of CuI and an unmodified Cu foil are plotted correspondingly in (A) and (B) for a comparison.

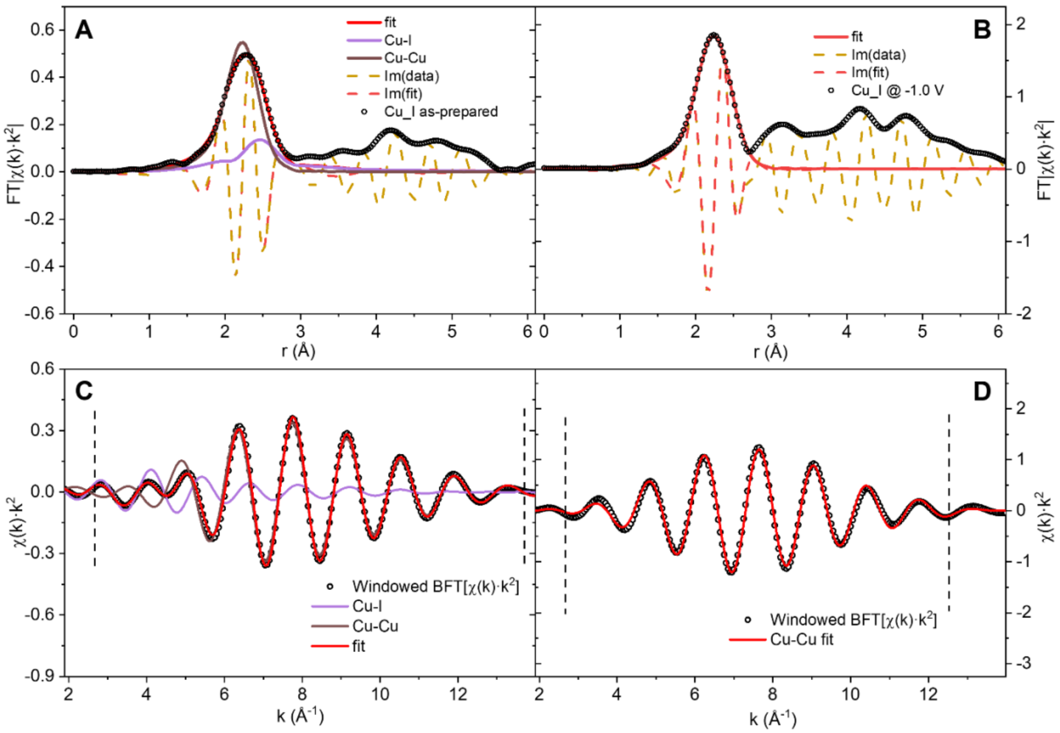

Figure S4. EXAFS spectra fitting of the Cu_I sample as-prepared (A) and (C); and under CO_2RR conditions after 1 h reaction at −1.0 V vs RHE in 0.1M $KHCO_3$ (B) and (D). Fitting results are represented by raw windowed reverse Fourier-transformed EXAFS spectra (open circles); fitting model (red lines) and the corresponding imaginary parts (dashed dark yellow and red lines). Cu-I and Cu-Cu paths are also shown (violet and dark red lines correspondingly).

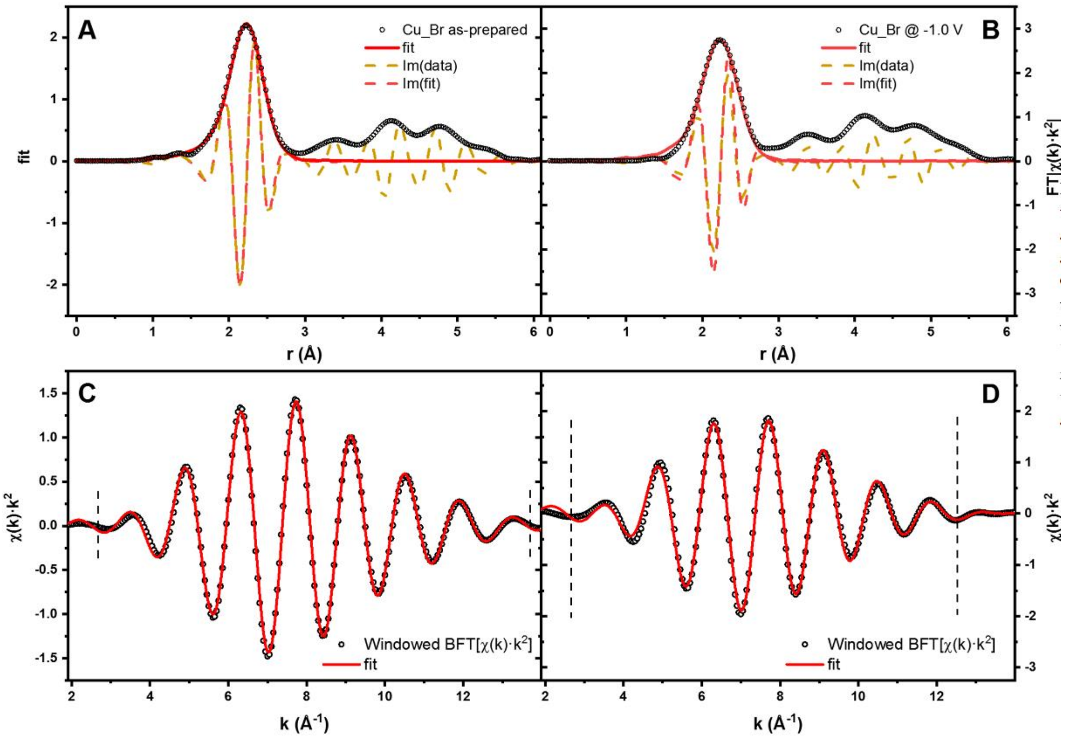

Figure S5. EXAFS spectra fitting of the Cu_Br sample as-prepared (A) and (C); and under CO_2RR conditions after 1 h reaction at −1.0 V vs RHE in 0.1M $KHCO_3$ (B) and (D). Fitting results are represented by raw windowed reverse Fourier-transformed EXAFS spectra (open circles); fitting model (red lines) and the corresponding imaginary parts (dashed dark yellow and red lines).

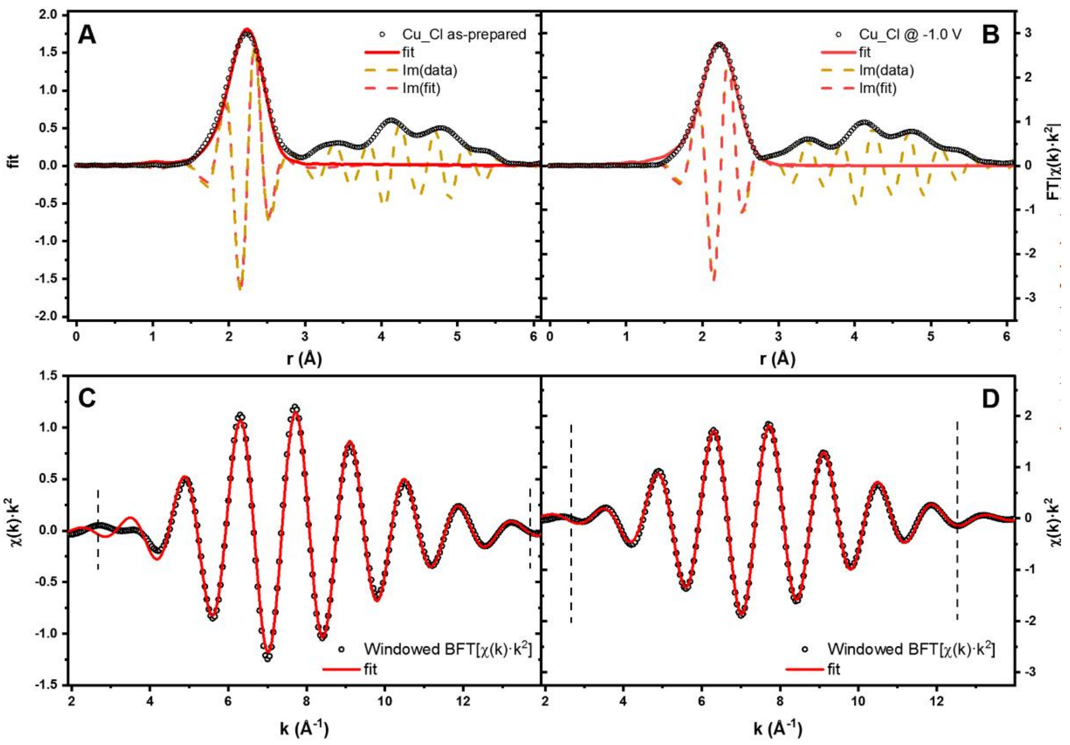

Figure S6. EXAFS spectra fitting of the Cu_Cl sample as-prepared (A) and (C); and under CO_2RR conditions after 1 h reaction at −1.0 V vs RHE in 0.1M $KHCO_3$ (B) and (D). Fitting results are represented by raw windowed reverse Fourier-transformed EXAFS spectra (open circles); fitting model (red lines) and the corresponding imaginary parts (dashed dark yellow and red lines).

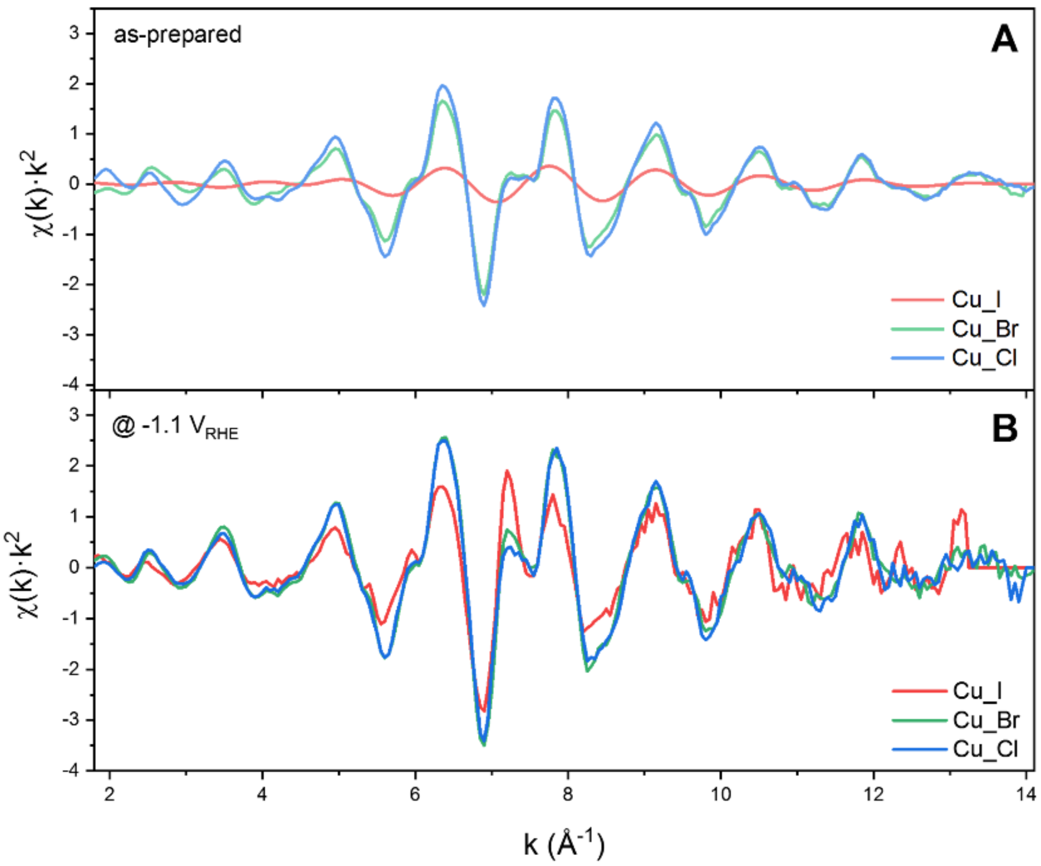

Figure S7. Raw $\chi(k)\cdot k^2$ EXAFS spectra of Cu_I, Cu_Br and Cu_Cl samples as-prepared (A) and under reaction conditions after 1 h reaction at −1.0V vs RHE in 0.1M $KHCO_3$ (B).

Table S4. Best-fit parameters of the Cu K-edge EXAFS spectra shown in Figure S4. Included are coordination numbers (CN), interatomic distances (*r*) and Debye-Waller factors (σ^2). The values in the parenthesis are the standard errors in the last digit. Reference *CN* and *r*-values are presented for a comparison.

Sample		Cu-Cu				Cu-X				R-factor
		CN	r, Å	$\sigma^2 \cdot 10^3$, $Å^{-2}$	ΔE, eV	CN	r, Å	$\sigma^2 \cdot 10^3$, $Å^{-2}$	ΔE, eV	
**Cu_I**	as-prepared	2.7(2)	2.52(1)	6(1)	3.3(7)	1.0(3) CuI	2.61(3)	13(1)	3.8(3)	0.008
	CO_2RR	8.5(5)	2.55(1)	6(1)	2.7(8)	-	-	-	-	0.014
**Cu_Br**	as-prepared	8.3(6)	2.53(1)	7.0(8)	3.1(9)	-	-	-	-	0.008
	CO_2RR	11(1)	2.53(1)	6.0(1)	3.5(4)	-	-	-	-	0.010
**Cu_Cl**	as-prepared	7.5(9)	2.53(1)	6.1(1)	1.1(4)	-	-	-	-	0.008
	CO_2RR	8.8(7)	2.53(1)	5.7(8)	2.8(3)	-	-	-	-	0.012
**CuI**		-	-	-	-	4	2.62	-	-	-
**Bulk Cu**		12	2.55	-	-	-	-	-	-	-

The EXAFS data of the as-prepared Cu_Br and Cu_Cl samples show close resemblance to metallic Cu, with an intense backscattering event at 2.25 Å (phase shift uncorrected, Figure S3-A). Their intensity, however, is lower than that of the bulk Cu reference spectrum, indicating a defective structure rich in undercoordinated Cu sites and/or larger structural disorder. EXAFS fitting revealed Cu-Cu coordination numbers (CNs) of 8.3 and 7.5 for Cu_Br and Cu_Cl, correspondingly (Table S4), while CN = 12 is expected for a face-centered Cu structure. The spectrum of the as-prepared Cu_I is significantly less intense, broader and shifted to 2.3 Å (uncorrected). Typically, various neighbors contributing to the first-shell peak can explain such a behavior, and indeed, the spectrum cannot be fitted with a Cu-Cu contribution alone, but requires an addition of Cu-I (Figure S4-A). After 1 h of CO_2RR, all three samples must still have defective surfaces as indicated by the low magnitude of the EXAFS spectra (Figure S3-B), and the corresponding Cu-Cu coordination numbers obtained (10.5, 8.8, 8.5 for Cu_Br, Cu_Cl and Cu_I respectively).

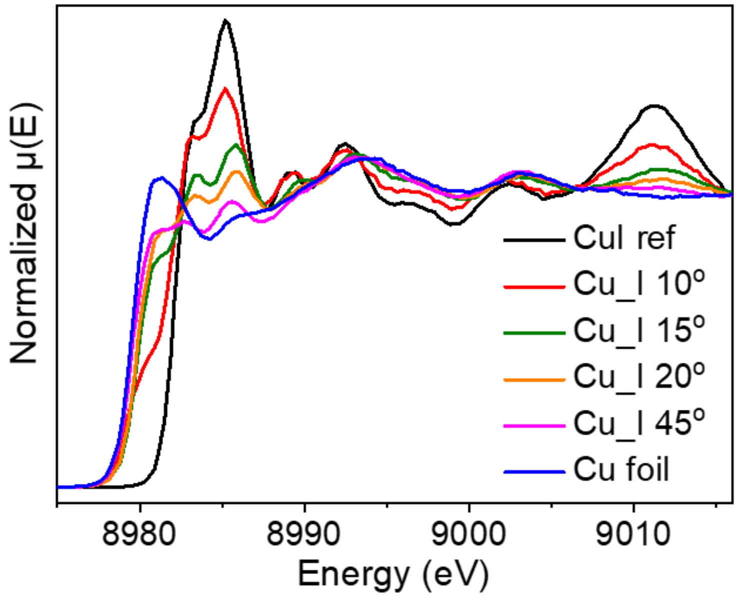

Figure S8. HERFD-XANES spectra of the as prepared Cu_I sample collected at different incidence angles. Bulk Cu and CuI spectra are plotted as reference.

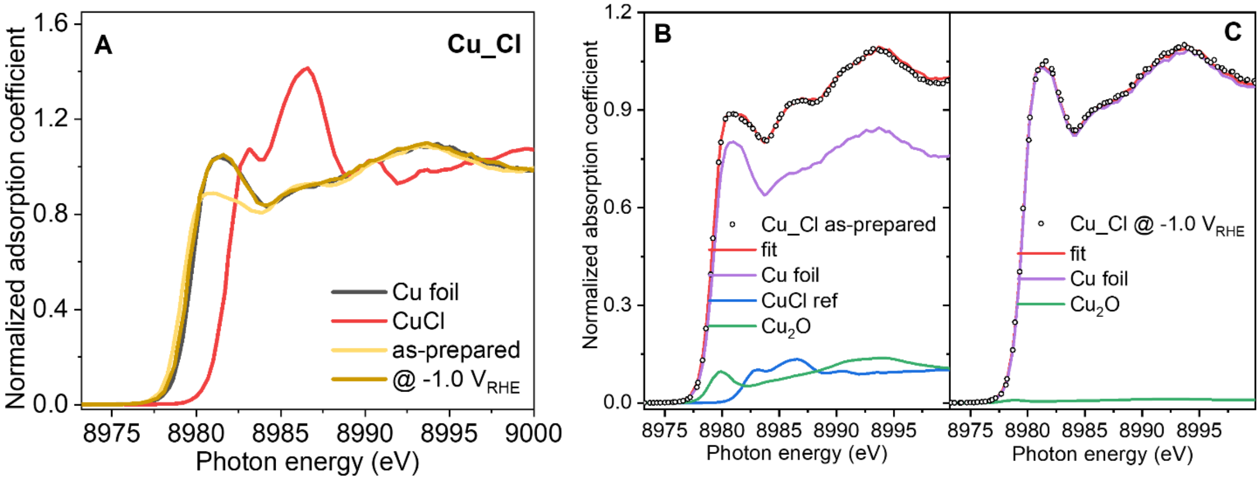

Figure S9. (A) HERFD-XANES spectra of the Cu_Cl sample. Samples were measured as-prepared and under *operando* conditions in 0.1M $KHCO_3$ after 1 h of CO_2RR at −1.0 V vs RHE. Linear combination analysis (LCA) of the as-prepared sample (B) and under *operando* conditions (C), the reference spectra of Cu foil, CuCl and Cu_2O are scaled according to their weighting parameters.

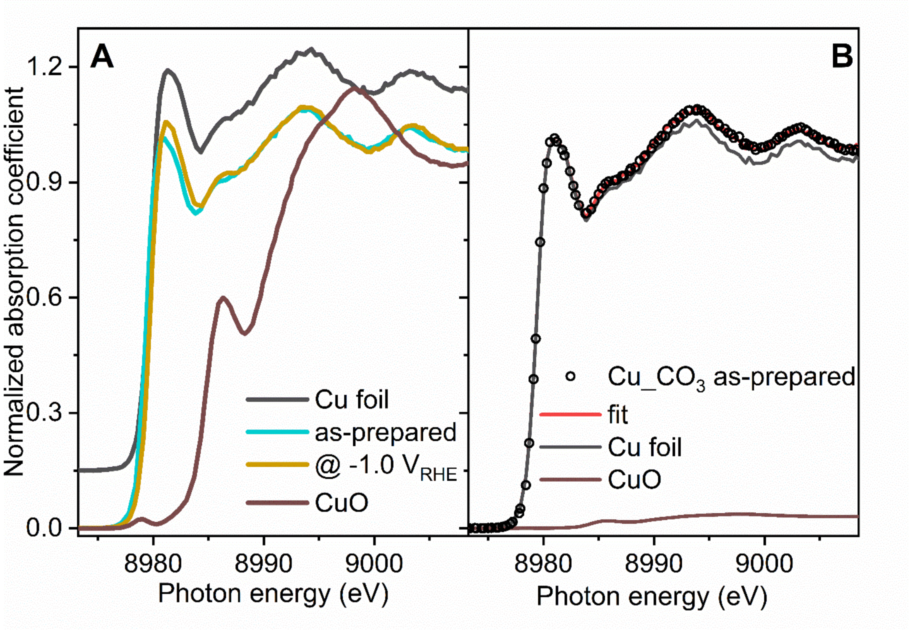

Figure S10. (A) HERFD-XANES spectra of the Cu_CO_3 sample. Samples were measured as-prepared and under *operando* conditions in 0.1M $KHCO_3$ after 1 h of CO_2RR at −1.0 V vs RHE. (B) Linear combination analysis (LCA) of the as-prepared sample, the reference spectra of Cu foil and CuO are scaled according to their weighting parameters.

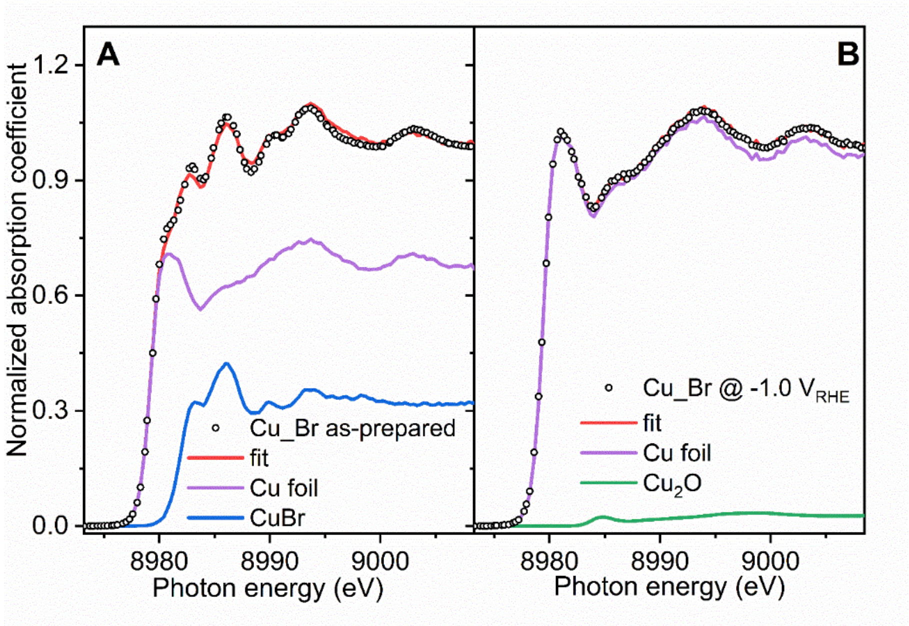

Figure S11. Linear combination analysis (LCA) of HERFD-XANES spectra of the Cu_Br sample as-prepared (A) and measured under *operando* conditions in 0.1M $KHCO_3$ after 1 h of CO_2RR at −1.0 V vs RHE (B). The reference spectra of a Cu foil, CuBr and Cu_2O are scaled according to their weighting parameters.

Table S5. Composition of Cu_Cl, Cu_Br, Cu_I, and Cu_CO_3 as-prepared and under CO_2RR at −1.0 V vs RHE in 0.1 M $KHCO_3$, as assessed by linear combination analysis (LCA) of HERFD XANES spectra. The CuX column shows the values of the corresponding halide spectra – CuI, CuBr and CuCl.

**Sample**		**Cu**		**Cu_xO**		**CuX**		R-factor
		X, at %	ΔE, eV	X, at %	ΔE, eV	X, at %	ΔE, eV	
**Cu_I**	as-prepared	37(1)	−0.53(4)	-	-	63(1)	0.03(1)	0.0011
	CO_2RR	86(2)	−0.15(2)	8(2) Cu_2O	−0.15(2)	7(1)	−3.1(1)	0.0006
**Cu_Br**	as-prepared	68(1)	−0.53(2)	-	-	32(1)	0.07(3)	0.0005
	CO_2RR	97(1)	−0.30(1)	3(1) Cu_2O	2.9(2)	-	-	0.0002
**Cu_Cl**	as-prepared	76.1(2)	−0.50(2)	14(1) Cu_2O	−1.8(2)	10(2)	0.07(1)	0.0003
	CO_2RR	99(1)	−0.31(1)	1.0(4) Cu_2O	−3.0(8)	-	-	0.0003
**Cu_CO_3**	as-prepared	97(1)	−0.36(1)	3(1) CuO	3.3(2)	-	-	0.0002
	CO_2RR	100	−0.02	-	-	-	-	0.0002

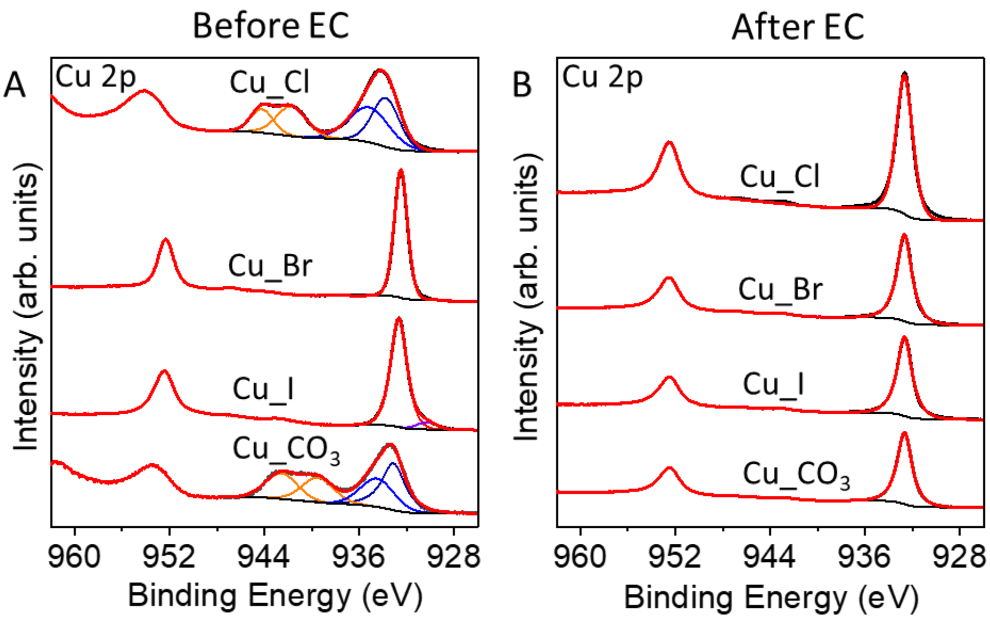

Figure S12. Quasi *in situ* Cu 2p XPS spectra of Cu_CO_3, Cu_Br and Cu_I before (A) and after (B) 1 h of CO_2RR at −1.0 V vs RHE in a CO_2-saturated 0.1 M $KHCO_3$ solution.

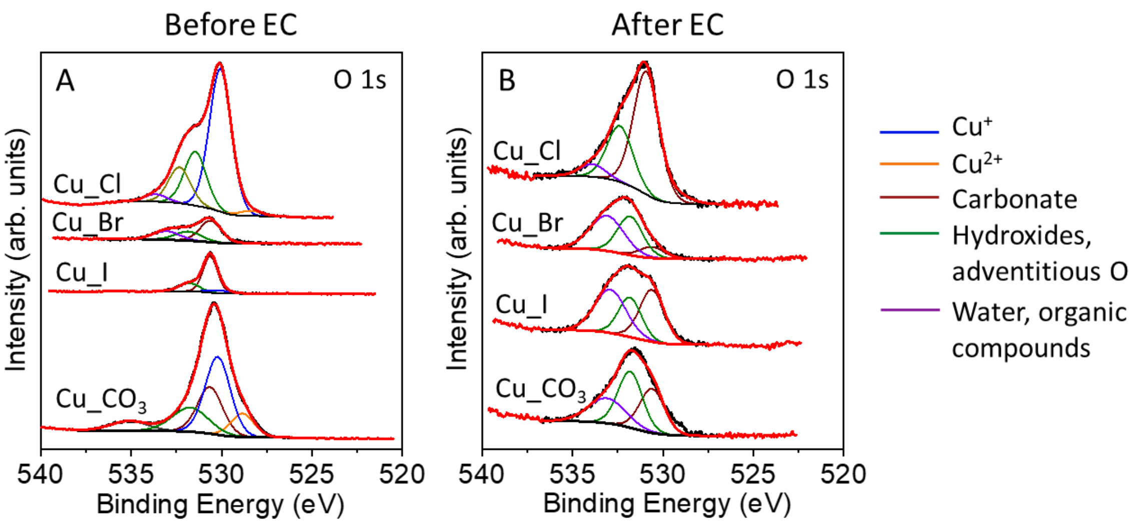

Figure S13. Quasi *in situ* O 1s XPS spectra of Cu_CO_3, Cu_Br and Cu_I before (A) and after (B) 1 h of CO_2RR at −1.0 V vs RHE in a CO_2-saturated 0.1 M $KHCO_3$ solution.

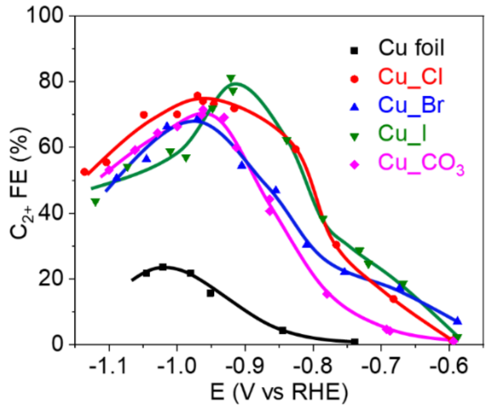

Figure S14. Total Faradaic efficiency of C_{2+} products as a function of the applied potential after 1 h of CO_2RR in a CO_2-saturated 0.1 M $KHCO_3$ solution.

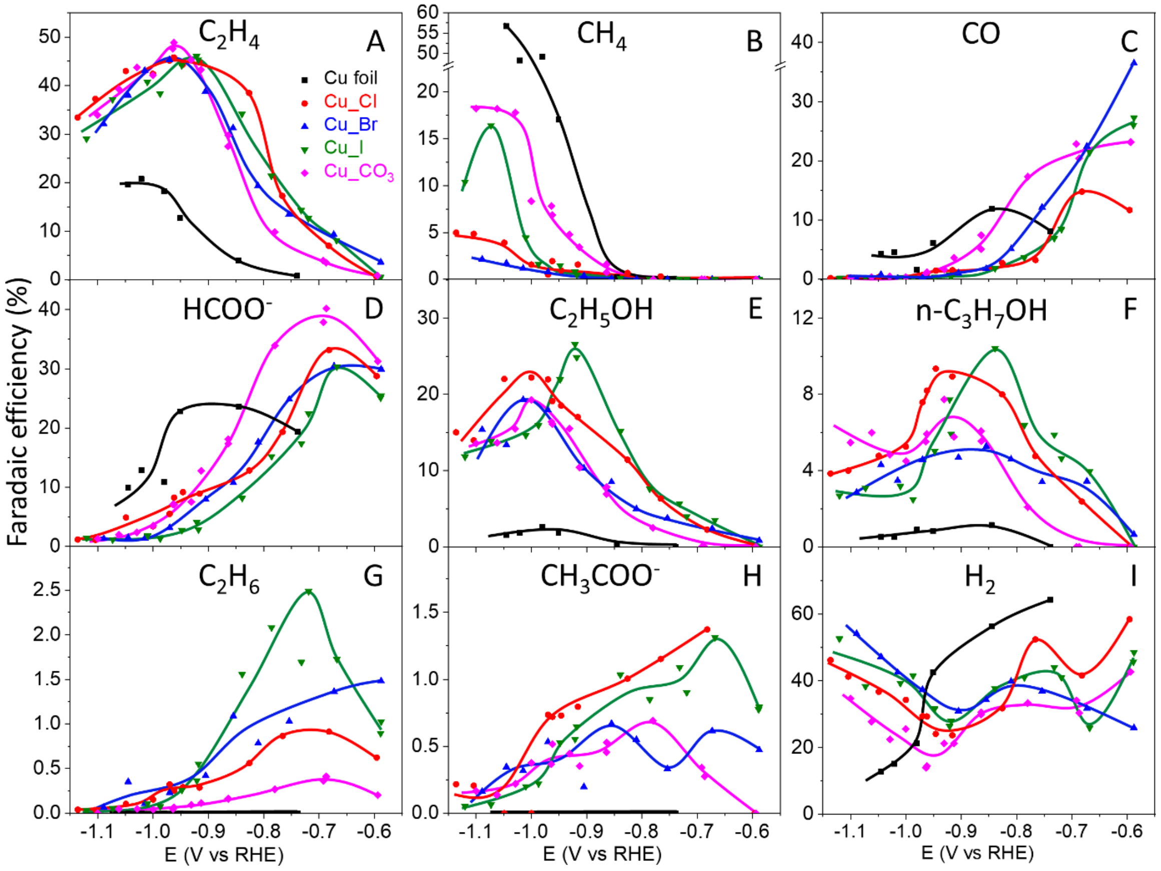

Figure S15. Faradaic efficiencies of C_2H_4 (A), CH_4 (B), CO (C), $HCOO^-$ (D), C_2H_5OH (E), n-C_3H_7OH (F), C_2H_6 (G), CH_3COO^- (H), and H_2 (I) as a function of the applied potential after 1 h of CO_2RR in a CO_2-saturated 0.1 M $KHCO_3$ solution. Solid lines are guides for the eye.

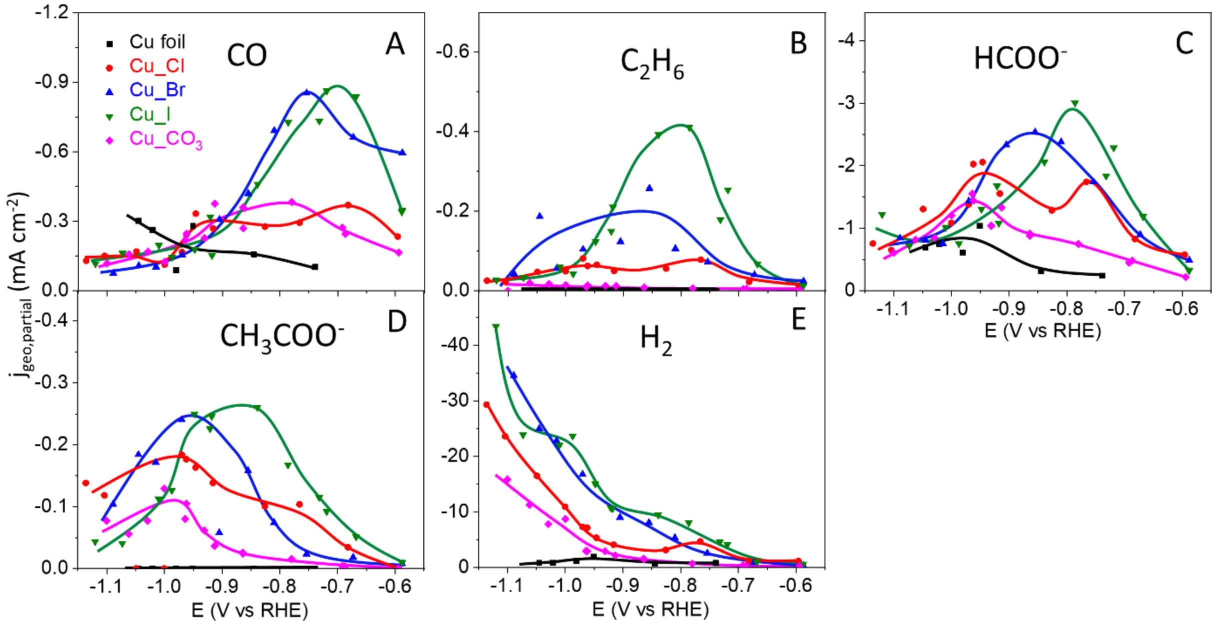

Figure S16. Geometric partial current densities of CO (A), C_2H_6 (B), $HCOO^-$ (C), CH_3COO^- (D), and H_2 (E) as a function of the applied potential after 1 h of CO_2RR in a CO_2-saturated 0.1 M $KHCO_3$ solution. Solid lines are guides for the eye.

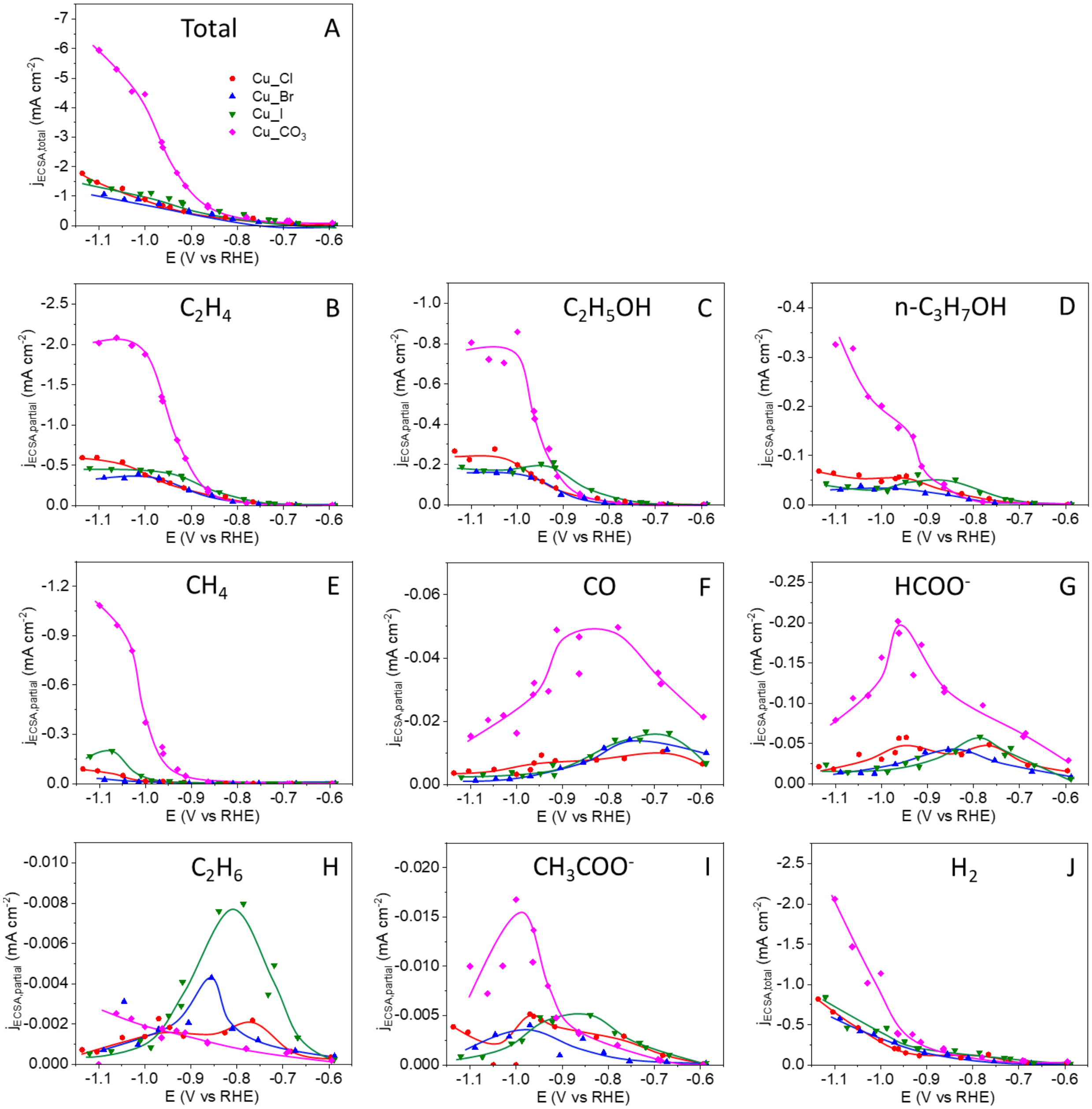

Figure S17. ECSA-normalized total (A) and partial current densities of C_2H_4 (B), C_2H_5OH (C), n-C_3H_7OH (D), CH_4 (E), CO (F), $HCOO^-$ (G), C_2H_6 (H), CH_3COO^- (I) and H_2 (J) as a function of the applied potential after 1 h of CO_2RR in a CO_2-saturated 0.1 M $KHCO_3$ solution. Solid lines are guides for the eye.

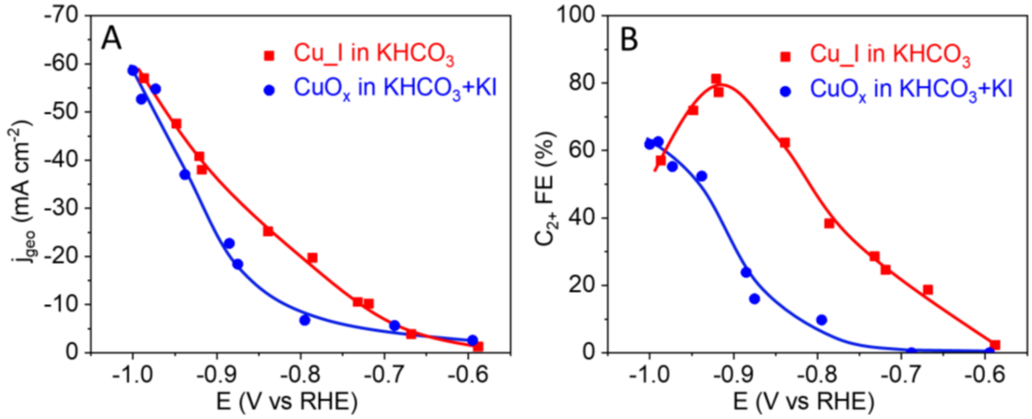

Figure S18. Potential-dependent geometric current density (A) and C_{2+} Faradaic efficiency (B) of the Cu_I catalyst measured in 0.1 M $KHCO_3$ in this work versus the O_2-plasma activated CuO_x catalyst measured in 0.1 M $KHCO_3$ + 0.3 M KI from our previous work[4].

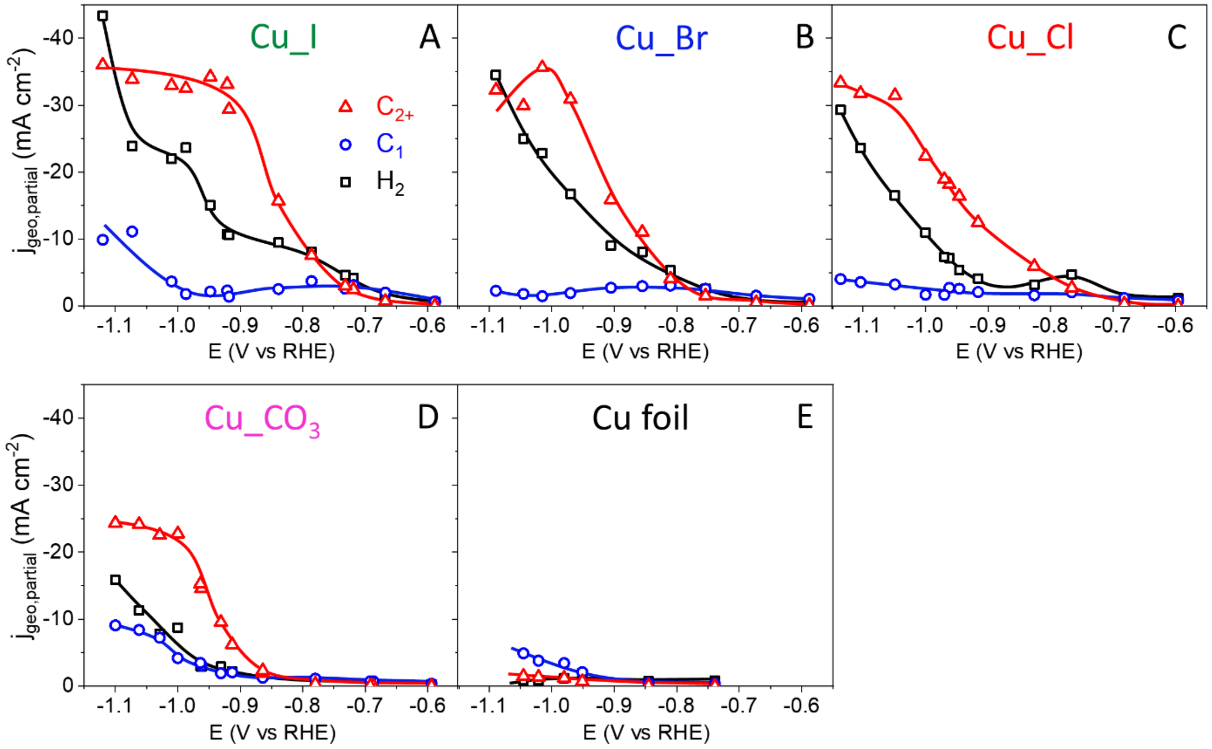

Figure S19. Geometric partial current densities of C_{2+}, C_1 and H_2 over (A) Cu_I, (B) Cu_Br, (C) Cu_Cl , (D) Cu_CO_3, and (E) Cu foil, as a function of applied potential after 1 h of CO_2RR in a CO_2-saturated 0.1 M $KHCO_3$ solution. Solid lines are guides for the eye.

Table S6. Maximum C_{2+} FE and corresponding geometric C_{2+} partial current densities of Cu catalysts measured in a 0.1 M $KHCO_3$ solution in an H-cell in this work and in the literature.

Catalyst	Potential / Current density	C_{2+} FE (%)	Partial current density for C_{2+} (mA cm^{-2})	Ref.
Cu_I	−0.9 V vs RHE	80	31.2	This work
Cu_Br	−1.0 V vs RHE	68	33.2	This work
Cu_Cl	−1.0 V vs RHE	73	20.7	This work
Cu_CO_3	−0.95 V vs RHE	71	14.9	This work
Cu nanocube	−0.963 V vs RHE	~60	~40	5
Electrochemically cycled Cu	−1.0 V vs RHE	~56	~5	6
Oxide-derived Cu	−0.95 V vs RHE	14	2.8	7
Cu_2O-derived Cu films	−31.2 mA cm^{-2}	59.8	18.7	8
Cu_2O film	−0.99 V vs RHE	49	12.3	9
CuCl-derived Cu	−2.6 V vs Ag/AgCl	73	12.4	10
electro-redeposited (ERD) Cu	−1.2 V vs RHE	52	31	11
Plasma-Cu foil	−1.0 V vs RHE	64.6	18.3	12
$Cu_{1.8}Se$ NW	−1.1 V vs RHE	79	11.5	13
Cu Mesh Catalysts	−1.1 V vs RHE	44	5.5	14
B-doped Cu	−1.1 V vs RHE	79 ± 2	55	15
Copper(II) phthalocyanine	−0.86 V vs RHE	13	~2	16

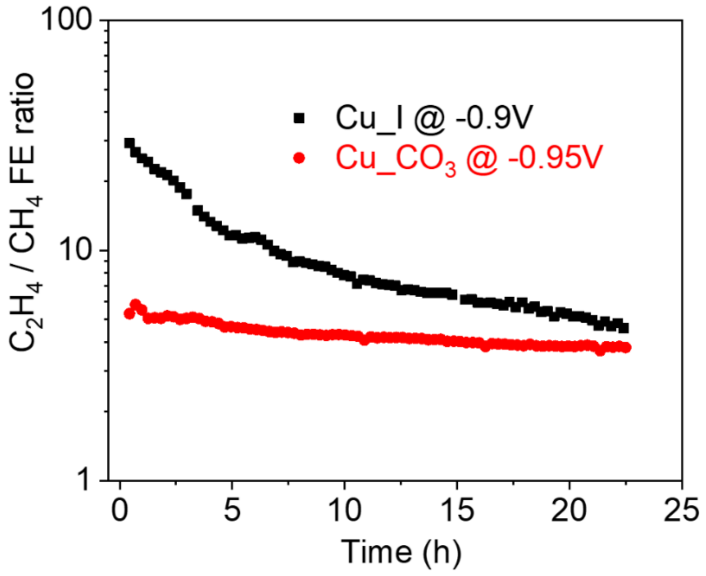

Figure S20. Time-dependent C_2H_4/CH_4 FE ratio over Cu_I at −0.9 V vs RHE and Cu_CO_3 at −0.95 V vs RHE in a CO_2-saturated 0.1 M $KHCO_3$ solution.

References:

1. B. Ravel, M. Newville, *J. Synchrotron Radiation* **2005**, *12*, 537–541.
2. J.J. Rehr, J.J. Kas, M.P. Prange, A.P. Sorini, Y. Takimoto, F.D. Vila, *Comptes Rendus Physique* **2009**, *10*, 548–559.
3. R.W.G. Wyckoff, E. Posnjak, *J. Am. Chem. Soc.* **1922**, *44*, 30–36.
4. D. Gao, F. Scholten, B. Roldan Cuenya, *ACS Catal.* **2017**, *7*, 5112–5120.
5. K. Jiang, R. B. Sandberg, A. J. Akey, X. Liu, D. C. Bell, J. K. Nørskov, K. Chan, H. Wang, *Nat. Catal.* **2018**, *1*, 111–119.
6. Y. Lum, B. Yue, P. Lobaccaro, A. T. Bell, J. W. Ager, *J. Phys. Chem. C* **2017**, *121*, 14191–14203.
7. C. W. Li, M. W. Kanan, *J. Am. Chem. Soc.* **2012**, *134*, 7231–7234.
8. A. D. Handoko, C. W. Ong, Y. Huang, Z. G. Lee, L. Lin, G. B. Panetti, B. S. Yeo, *J. Phys. Chem. C* **2016**, *120*, 20058–20067.
9. D. Ren, Y. Deng, A. D. Handoko, C. S. Chen, S. Malkhandi, B. S. Yeo, *ACS Catal.* **2015**, *5*, 2814–2821.
10. G. Kibria, C. Dinh, A. Seifitokaldani, P. De Luna, T. Burdyny, R. Quintero-bermudez, M. B. Ross, O. S. Bushuyev, F. P. G. De Arquer, P. Yang, et al., *Adv. Mater.* **2018**, *30*, 1804867.
11. P. De Luna, R. Quintero-Bermudez, C.-T. Dinh, M. B. Ross, O. S. Bushuyev, P. Todorović, T. Regier, S. O. Kelley, P. Yang, E. H. Sargent, *Nat. Catal.* **2018**, *1*, 103–110.
12. D. Gao, I. T. McCrum, S. Deo, Y. W. Choi, F. Scholten, W. Wan, J. G. Chen, M. J. Janik, B. Roldan Cuenya, *ACS Catal.* **2018**, *8*, 10012–10020.
13. Y. Mi, X. Peng, X. Liu, J. Luo, *ACS Appl. Energy Mater.* **2018**, *1*, 5119–5123.
14. M. Rahaman, A. Dutta, A. Zanetti, P. Broekmann, *ACS Catal.* **2017**, *7*, 7946–7956.
15. Y. Zhou, F. Che, M. Liu, C. Zou, Z. Liang, P. De Luna, H. Yuan, J. Li, Z. Wang, H. Xie, et al., *Nat. Chem.* **2018**, *10*, 947–980.
16. Z. Weng, Y. Wu, M. Wang, J. Jiang, K. Yang, S. Huo, X. Wang, Q. Ma, G. W. Brudvig, V. S. Batista, et al., *Nat. Commun.* **2018**, *9*, 415.
